# Volumetric Changes in the Basal Ganglia After Antipsychotic Monotherapy: A Systematic Review

**DOI:** 10.2174/0929867311320030015

**Published:** 2013-01

**Authors:** B.H Ebdrup, H Nørbak, S Borgwardt, B Glenthøj

**Affiliations:** 1 Center for Neuropsychiatric Schizophrenia Research, CNSR & Center for Clinical Intervention and Neuropsychiatric Schizophrenia Research, CINS, Copenhagen University Hospital, Psychiatric Center Glostrup, Denmark; 2 Department of Psychiatry, Medical Image Analysis Center, University of Basel, Switzerland

**Keywords:** Basal ganglia; first generation antipsychotic; magnetic resonance imaging; monotherapy; psychosis; schizophrenia; second generation antipsychotic

## Abstract

**Introduction:**

Exposure to antipsychotic medication has been extensively associated with structural brain changes in the basal ganglia (BG). Traditionally antipsychotics have been divided into first and second generation antipsychotics (FGAs and SGAs) however, the validity of this classification has become increasingly controversial. To address if specific antipsychotics induce differential effects on BG volumes or whether volumetric effects are explained by FGA or SGA classification, we reviewed longitudinal structural magnetic resonance imaging (MRI) studies investigating effects of antipsychotic monotherapy.

**Material and Methods:**

We systematically searched PubMed for longitudinal MRI studies of patients with schizophrenia or non-affective psychosis who had undergone a period of antipsychotic monotherapy. We used specific, predefined search terms and extracted studies were hand searched for additional studies.

**Results:**

We identified 13 studies published in the period from 1996 to 2011. Overall six compounds (two classified as FGAs and four as SGAs) have been investigated: haloperidol, zuclophentixol, risperidone, olanzapine, clozapine, and quetiapine. The follow-up period ranged from 3-24 months.

Unexpectedly, no studies found that specific FGAs induce significant BG volume increases. Conversely, both volumetric increases and decreases in the BG have been associated with SGA monotherapy.

**Discussion:**

Induction of striatal volume increases is not a specific feature of FGAs. Except for clozapine treatment in chronic patients, volume reductions are not restricted to specific SGAs.

The current review adds brain structural support to the notion that antipsychotics should no longer be classified as either FGAs or SGAs. Future clinical MRI studies should strive to elucidate effects of specific antipsychotic drugs.

## 1 INTRODUCTION

**Fig. (1):**
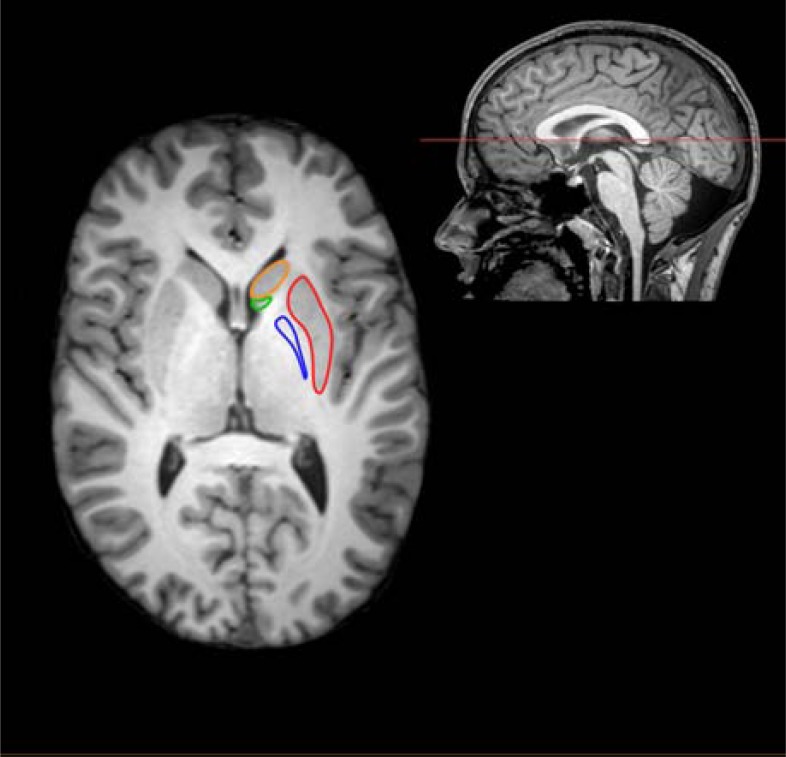
Anatomical localization of the basal ganglia (BG) structures is based on a high-resolution T1-weighted magnetic resonance image acquired on a Philips Achieva 3.0T whole body MRI scanner (Philips Healthcare, Best, The Netherlands) with a 32-channel head coil. The colored circles indicate the regions of interest (ROIs) comprising the basal ganglia (BG): [orange: caudate nucleus]; [green: nucleus accumbens]; [red: putamen]; [blue: globus pallidus]. Functionally, the basal ganglia (BG) structures are particularly involved in two major dopaminergic pathways [68]:
The mesolimbic pathway: From the ventral tegmentum dopamine is transmitted to the nucleus accumbens and the ventral part of caudate nucleus (the associative part of striatum).The nigro-striatal pathway: from substantia nigra dopamine is transmitted to the posterior part of the caudate nucleus and putamen (areas primarily involved in motor control).

Schizophrenia is a severe and heterogeneous brain disease which is associated with progressive and widespread structural brain changes [1-3]. At the same time antipsychotics, which constitute the cornerstone in the treatment of schizophrenia and other psychoses, also affect brain structure. Owing to the burgeoning interest for magnetic resonance imaging (MRI) and the continuous refinement of these techniques it is now evident that both global brain measures (e.g. total gray matter or total brain volume) [4,5] and more regional brain structures (e.g. temporal lobe or subcortical structures) may be affected by antipsychotic medication [6]. A shared feature of all current antipsychotic compounds is an affinity for the dopamine D_2_ receptors which are highly expressed in the basal ganglia (BG) [7]. The BG comprises the caudate nucleus, nucleus accumbens, putamen and globus pallidus, as illustrated in (Fig. **1**). For this reason the BG has drawn particular interest in the pursuit of disentangling associations between antipsychotic medication and structural brain changes. 

The first observations of volumetric aberrations in the BG in antipsychotic treated patients were of volumetric increases [8-11]. This increase was proposed to reflect the degree of dopaminergic blockade in BG [9,10]. The notion that antipsychotics can induce volume increases in BG has later been confirmed in several preclinical studies [12-14]. Nevertheless, other pre-clinical studies using haloperidol have not replicated these BG volume increases [15,16]. Also, clozapine, which is a relatively weak D_2_ receptor antagonist, has been associated with BG volume increases [13]. 

The trajectory and timing of the effects of antipsychotics on structural brain changes is unclear. An understanding of the trajectory of brain changes is particularly relevant in the context of the neurodevelopmental course of psychosis. A number of longitudinal structural neuroimaging studies have found progressive brain changes in adults with schizophrenia during the initial years after the onset of the illness [17]. The recent development of the clinical high risk (HR) strategy has allowed investigations of structural and functional alterations in un-medicated patients before the onset of the illness. These neuroimaging studies have shown alterations in brain structure [18,19], function [20], and chemistry [21,22] that are largely comparable between chronic patients and un-medicated HR subjects of psychosis. Clinically, these early progressive brain changes are associated with poorer outcomes, more negative symptoms, and a decline in neuropsychological performance [23]. It is therefore pertinent to unravel to what extend structural brain changes are associated with antipsychotic medication.

Early preclinical studies reported changes in BG already after 1 month and that these where sustained after 12 months [12]. In longitudinal clinical MRI studies changes in the basal ganglia have been reported after treatment periods ranging from 3 weeks, e.g. [24] to 7.2 years [5]. Interestingly, a recent study in healthy volunteers showed a reversible volumetric *decrease* in the caudate nucleus already 1-2 hours after haloperidol injection (5 mg/70 kg) [25]. Clinically, the BG volume increases may be dose-dependent [14] and possibly associated with more extrapyramidal symptoms (EPS) [12,25].

Initially the classification of antipsychotics into so-called ‘typical’ and ‘atypical’ compounds was based on their tendency to induce extrapyramidal side effects, e.g. tardive dyskinesia [26,27]. Later, the terms first and second generation antipsychotics (FGAs and SGAs) have been more frequently used (and this terminology will be used throughout the present review). 

Several systematic reviews of clinical MRI studies has led to the prevailing assumption that FGAs induce BG volume increases and that SGAs either has no volumetric effect or reduces the BG volume [6,28-30]. Since most clinical studies have grouped patients based on either FGA or SGA treatment this may impose a limitation to this dogma. From a pharmacological perspective there is an immense variation between antipsychotic drugs both regarding receptor profiles and pharmacokinetic aspects (e.g. half-lives and affinities for individual receptor systems and subtypes) [31]. In recent years it has become evident that individual antipsychotic compounds, regardless of FGA or SGA classification, also differ considerably with respect to side-effect profiles and to a certain degree on clinical efficacy [32-35]. Consequently, it has been proposed that the classification of FGAs and SGAs should no longer be upheld [32,35].

The abortion of this pragmatic dichotomy between FGAs and SGAs implies that the paradigm of the proposed differential effect on brain structure needs to be revisited. To address if specific antipsychotics induce differential effects on BG volumes or whether volumetric effects are explained by FGA or SGA classification, we here review longitudinal MRI studies involving antipsychotic monotherapy in patients with a diagnosis in the schizophrenia spectrum.

## 2 MATERIALS AND METHODS

We conducted a systematic review on clinical longitudinal structural MRI studies of patients with a diagnosis of schizophrenia or non-affective psychosis who had undergone a period of antipsychotic monotherapy. A clear specification of the antipsychotic compound used and the treatment period was required. The medication doses prior to inclusion (exposure time and dosage e.g. in chlorpromazine equivalents) and during the intervention were recorded when available. Information about patients’ previous anti-Psychotic exposure (prior to entering the studies) was used to evaluate potentially confounded volumetric observations. Likewise, information on substance abuse was noted when available. In the current study the term ‘monotherapy’ specifically refers to ‘anti-psychotic monotherapy’. Studies in which concomitant medication, e.g. anticholinergics or benzodiazepines, had been used were not excluded. Both studies investigating first-episode and chronic patients were included. Only studies examining a predefined region of interest (ROI) comprising of any combination of the structures in the basal ganglia, i.e.: striatum (comprising caudate nucleus, nucleus accumbens and putamen) and globus pallidus were included. 

We searched PubMed [36] using the MeSH terms ‘schizophrenia’; ‘magnetic resonance imaging’; ‘antipsychotic agents’; ‘basal ganglia’. Moreover, we conducted an unrestricted PubMed search including the terms: ‘psychosis’; ‘brain structure’; ‘subcortical’; ‘striatum’; ‘caudate’; ‘accumbens’; ‘putamen’; ‘globus pallidus’; ‘neuroleptic’; ‘monotherapy’; ‘first generation’; ‘second generation’; ‘typical’; ‘atypical’. Finally, extracted studies were hand searched for additional references. PubMed search dates were from 1984 (were the first MRI study was performed [37]) until June 2012. Only articles written in English were included. Observations of volumetric associations with clinical variables such as symptoms (e.g. positive and negative syndrome scale (PANSS) scores), level of functioning (e.g. clinical gobal impression (CGI) scores), and side-effects (e.g. extrapyramidal side-effects (EPS)) were also assessed when available. 

*A priori* we assigned highest priority to significant findings based on the following two types of analyses commonly used to report volumetric changes in a patient group: 1) numerical volumes at baseline contrasted to numerical volumes at follow-up within a patient group [(baseline volume x follow-up volume)_patients_]; 2) volumetric changes in a patient group contrasted to volumetric changes in a healthy control group [(baseline volume – follow-up volume)_patients_ x (baseline volume – follow-up volume)_controls_]. Reports of *relative* volumetric changes between two patient groups including two different treatment regimens (e.g. volumetric changes in an olanzapine treated group vs. changes in a haloperidol group) were assigned less emphasis since interaction effects may obscure the effects of monotherapy. 

## 3 RESULTS

**Fig. (2):**
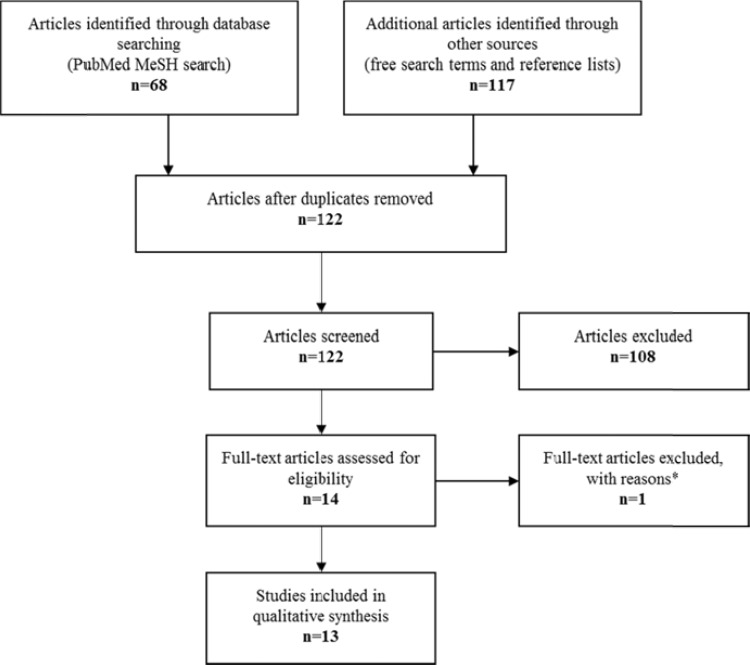
Flowchart of the literature search. The flowchart is adapted from [69]. *The study by Arango *et al*. 1992 [52] was excluded because of an unclear period of monotherapy (comprising a 4-week phase in which patients were switched from fluphenazine (10-30mg/d) to either clozapine or haloperidol treatment). Moreover, at follow-up only a cross-sectional statistical test between the two patient groups was reported. The volumetric data on the controls was identical to that presented in the baseline study [8]. Finally the time period and the antipsychotic intervention between the baseline and the follow-up MRI scans were judged insufficiently specified for the present purpose.

We identified 13 studies fulfilling our inclusion criteria. A flowchart of the selection procedure with the included and excluded studies is shown in (Fig. **2**). All studies were written in English and published between 1996 and 2011. Overall, six compounds (two classified as FGAs and four as SGAs) have been subject to investigation: haloperidol in 2 studies [4,38]; zuclophentizol in 1 study [39]; risperidone in 5 studies [38-42]; olanzapine in 4 studies [4,38,42,43]; clozapine in 4 studies [11,44-46]; and quetiapine in 2 studies [47,48]. The follow-up period ranged from 3-24 months.

In the following we summarize the findings of the 13 studies included. The text has been organized in separate sections for each of the six different compounds. Since we expected that volumetric changes in the BG would be associated with the dopaminergic potency, the ranking of the antipsychotics was based on the affinity to the D_2_ receptor starting with high affinity drugs [Table **1**]. Where several studies have investigated the same drug, the studies are presented chronologically. Because we *a priori* assigned less emphasis on the interaction between two different antipsychotic regimens, studies comprising several active treatment arms (e.g. haloperidol and olanzapine), were regarded as separate studies (i.e. data points) each representing one treatment arm. Thus, several studies are reported more times in the following. The same approach has been applied in (Table **2**) in which selected findings from the studies are shown as individual data points.

### 3.1 Haloperidol Monotherapy

*In 2005 Lieberman et al.* [4] published a longitudinal, randomized, controlled, multisite, double-blind study with 263 patients (aged 16 to 40 years) with a diagnosis of schizophrenia, schizophreniform, or schizoaffective disorder. Previous antipsychotic exposure did not exceed 16 cumulative weeks and none of patients had been treated with clozapine. Current substance abuse was an exclusion criterion. Patients were randomized to haloperidol (range: 2-20 mg/d) or olanzapine (range: 5-20 mg/d) for up to 24 months. Structural MRI scans were performed after 3, 6, 12 and 24 months. Healthy controls were only scanned at 3 and 12 months. In total seven ROIs were assessed and among these was the caudate nucleus. 

Remarkably, the caudate nucleus in the patients did not significantly increase after 3 or 12 months of haloperidol treatment as compared with the healthy controls. The number of patients and mean doses at these two time points were (n=68; 4.7 mg/d and n=31; 4.9 mg/d) [the mean doses were kindly provided after personal communication with the authors]. When compared to changes in the olanzapine group significant relative volumetric increases in the haloperidol group were observed at 6, 12 and 24 months. Nevertheless, these differences appeared driven by an *interaction* of volumetric changes between the two groups rather than by a clear cut increase in the haloperidol group (mean changes in caudate nucleus (in cm^3^): 6 months: -0.23 (Ola) vs. -0.04 (Hal); 12 months: -0.52(Ola) vs. 0.12 (Hal); -0.30 (Ola) vs. 0.39 (Hal). No associations with caudate nucleus volumes, medication and clinical response were observed.

*In 2008 Crespo-Facorro et al.* [38] published a longitudinal, randomized, open-label study with 183 patients (aged 15–60 years) with a diagnosis of schizophrenia, schizophreniform disorder,schizoaffective disorder, brief reactive psychosis, or psychosis not otherwise specified. Substance dependence was an exclusion criterion. All patients but one (who had been minimally medicated (<6 weeks)) were antipsychotic-naïve at the baseline MRI scanning. Patients were randomised to haloperidol (range: 3-9 mg/d), olanzapine (range: 5-20 mg/d), or risperidone (range: 3-6 mg/d). 

At baseline, 70 of the randomised patients underwent structural MRI scanning and 52 of these patients were re-scanned after 12 months. In the context of the present review, it should be noted that the study was only patially complying with our inclusion criterion of antipsychotic monotherapy: 8 of 18 patients in the haloperidol group; 4 of 18 patients in the olanzapine group and 4 of 18 patients in the risperidone group were “switched to different atypical antipsychotics during follow-up”. Since the authors stated that “the differences observed were not altered after controlling for the switch of medication during the study”, the study was still included here. In total twelwe ROIs were assessed, two of these were the caudate nucleus and putamen. 

Overall, 12 months of haloperidol treatment (n=18; 4.9 mg/d (mean doses calculated from [49]) did not significantly change the volumes of the caudate nucleus or putamen. In fact, 12 months of haloperidol treatment in an almost antipsychotic-naïve patient sample, numerically *reduced* the volumes of the caudate nucleus (-0.33%) and the putamen (-3.44%). To what extend these numerical volume reductions may be overestimated since 8 out of 18 of the patients had been switched to SGAs during the investigation period (mean time to switch: 6.4 months) is unclear.

*Post hoc* analyses of FGA (haloperidol) vs. SGAs (olanzapine and risperidone) revealed a significant time-by-group interaction in the volume of the caudate nucleus (p=.045). This difference was driven by a significant decrease in caudate nucleus volume (−3.10%, p=.001) in the SGA group as compared with the marginally reduced volume (i.e. a relative increase) in the haloperidol group (-0.33%). No associations with caudate nucleus or putamen volumes, medication and clinical response were observed.

### 3.2 Zuclopenthixol Monotherapy

*In 2007 Glenthøj et al.* [39] published a longitudinal, randomised, open-label study with 19 first-episode schizophrenia patients (mean age 26 years). Three patients had been minimally medicated before inclusion, but the results of the study remained unchanged if these subjects were excluded from the analyses. Fourteen patient had a history of substance abuse (alcohol n=2; cannabis n=10; alcohol and cannabis n=2). Patients were randomised to treatment for three months with either zuclopenthixol (n=8; dose range: 4-26 mg/d; mean dose 10.3 mg/d) or risperidone (n=11; dose range: 1-7 mg/d, mean dose 3.4 mg/d). Three ROIs were manually traced: the caudate nucleus, nucleus accumbens, and putamen. 

Three months treatment with zuclopenthizol did not significantly change the volumes of any of the striatal structures. Nevertheless, a crude numerical inspection of the striatal volumes indicate that all 3 structures marginally increased. No significant interactions between volumetric changes in the zuclopenthixol group and the risperidone group were observed. No associations with striatal volumes, medication and clinical response were observed.

### 3.3 Risperidone Monotherapy

*In 2001 Lang et al.* [41] published a longitudinal study with 30 first-episode patients (mean age 23 years) with a diagnosis of schizophrenia or schizoaffective disorder. Lifetime antipsychotic expoure did not exceed 8 weeks antipsychotic treatment the past year or 4 weeks of continous antipsychotic treatment prior to the baseline MRI scanning. A history of substance abuse was an exclusion criterion. Three ROIs were assessed: the caudate nucleus, putamen, and globus pallidus. Fifteen patients were re-scanned after 12 months of risperidone treatment (dose range 1-6 mg/d; mean dose 2.7 mg/d). At follow-up, no significant changes in the BG structures were observed in the patients. Also, no significant volumetric changes were observed in a group of healthy controls (n=23). No significant associations with striatal volumes, medication and clinical response were observed.

*In 2004 Lang et al.* [42] published a longitudinal study with 37 schizophrenia patients (mean age 23 years). The study included two patient groups, one group treated with FGAs (n=10) and one group treated with risperidone (n=27). After inclusion, patients in the risperidone group where either switched to olanzapine or continuing on risperidone during the following 12 months. The grouping was based on a clinical evaluation. The patients whom at baseline exhibited the most positive symptoms and most pronounced EPS despite risperidone treatment (mean dose 3.3 mg/d) were switched to olanzapine (n=13; mean dose 15.0 mg/d). The remaining patients with a satisfactory clinical response (n=14; mean dose 2.3 mg/d) continued risperidone treatment in a stable dosage (mean dose 2.1 mg/d). Three ROIs were assessed: the caudate nucleus, putamen, and globus pallidus. At follow-up no significant changes in any of the BG structures were observed and no interactions between the risperidone and the olanzapine groups emerged. Analyses of potential associations between striatal volumes, medication and clincial variables were not reported.

*In 2005 Massana et al.* [40] published a longitudinal study with 11 patients (age range 18-30 years; mean age 23 years) with a diagnosis of schizophrenia or schizophreniform disorder. All patients were first-episode and antipsychotic-naïve at the baseline MRI scanning. Data on substance abuse was not provided. In total ten ROIs were assessed and four of these were the caudate nucleus, nucleus accumbens, putamen, and globus pallidus. After 3 months of risperidone treatment (mean dose 6.05 mg/d) significant increases in left nucleus accumbens and left caudate nucleus were observed (based on corrected p-values). Uncorrected p-values p<0.001 (a less conservative statistical correction) revealed bilateral increases in all BG structures. Analyses of potential associations between ROI volumes, medication and clincial variables were not reported.

*In the 2007 study by Glenthøj et al.* [39] separate, *post hoc* analyses of the risperidone group revealed significant increases in the putamen after 3 months of risperidone treatment (mean dose 3.4 mg/d). Moreover, crude numerical inspection of the volumes indicate that also the remaining striatal structures (except for left nuclus accumens) marginally increased after 3 months of risperidone treatment.

*In the 2008 study by Crespo-Facorro et al.* [38] 12 months treatment with risperidone (n=16; mean dose 3.7 mg/d) did not significantly change the volumes of neither the caudate nucleus or the putamen. Numerically both structures were reduced in volume over time (-1.95% and -1.61%, respectively). 

### 3.4 Olanzapine Monotherapy

*In the 2004 study by Lang et al.* [42] two patient groups underwent olanzapine monotherapy. One group constituted patients who had responded unsatisfactory on the initial risperidone treatment. After 12 months of olanzapine treatment (n=13; mean dose 15.0 mg/d) no significant volumetric changes in the caudate nucleus, putamen, or globus pallidus were observed (see previous section on risperidone monotherapy for details). The other olanzapine monotherapy group included chronic patients (n=10) who had been treated with various FGAs, including loxapine, trifluoperazine, chlorpromazine, fluphenazine or haloperidol (mean dose 360.1 mg/d in chlorpromazine equivalents). After baseline MRI patients were switched to olanzapine for the next 12 months (mean dose 17.0 mg/d). At baseline, FGA treated patients had significant larger volumes of putamen (7.0%) and globus pallidus (20.7%) as compared with controls. After 12 months of olanzapine treatment the volumes of the putamen and globus pallidus had been reduced (by -9.8% and -10.7%, respectively) and they were no longer significantly different from putamen and globus pallidus volumes of the controls. Notably, no significant volumetric abberations were observed in the caudate nucleus, neither in the ‘FGA condition’ (at baseline) nor in the ‘olanzapine condition’ (at follow-up).

*In the 2005 study by Lieberman et al.* [4] the olanzapine treated patients (n=71; mean dose 9.5 mg/d; dose range: 5-20 mg/d) had a numerical, but non-significant volume reductions (-0.36 cm^3^; p=.18) in the caudate nucleus after 3 months as compared with controls. After 12 months (n=42) the caudate volume in patients was significantly reduced as compared with controls (-0.52 cm^3^ vs. 0.17 cm^3^; p=.003). For the interaction between the olanzapine and haloperidol groups, see the previous section on haloperidol monotherapy.

*In 2007 Okugawa* *et al.* [43] published a longitudinal, open-label study with 10 schizophrenia patients (mean age 31.6 years). Patients had a mean duration of illness of more than 4.7 years. Data on previous medication intake and substance abuse was not provided. After baseline MRI scanning patients were treated with olanzapine (mean dose 14.3 mg/d) until patients had a reduction in PANSS total score to less than 50% relative to the baseline values. Mean time to follow-up MRI scanning was 6 months. The volumes of gray and white matter in the caudate nucleus were the only ROI assessed. At baseline the volume of the caudate nucleus (both gray and white matter) in patients was significantly smaller than that of the controls. After 6 months of olanzapine treatment the volume of the caudate nucleus had significantly increased as compared with the volumes at baseline. The healthy controls were not re-scanned, but the baseline volumes of the gray matter in caudate nucleus in controls (2.80 ml) and the patients caudate volumes at follow-up (2.54 ml) did not differ significantly. Analyses of potential associations between caudate nucleus volumes, medication and clincial variables were not reported.

*In the 2008 study by Crespo-Facorro et al.* [38] 12 months treatment with olanzapine (n=18; mean dose 14.5 mg/d) did not significantly change the volumes of the caudate nucleus or the putamen. However, numerically both structures were reduced in volume over time (-4.10% and -3.18%, respectively).

### 3.5 Clozapine Monotherapy

*In 1995 Chakos et al.* [11] published a longitudinal study with 8 patients with chronic schizophrenia (mean age 31.4 years) who were schwitched to clozapine because of refractory symptoms or development of tardive dyskinesia. These eight patients were compared with 7 chronic schizophrenia patients (mean age 26.9) who continued FGA antipsychotic treatment. The compounds and doses of FGAs used were not specified. Data substanse abuse and clinical variables were not provided. The caudate nucleus was the only ROI assessed. Patients switched to clozapine had longer duration of illness (116 months) than patients in the FGA group (38 months). Patients were scanned at baseline and after approximately 12 months (54.6 weeks). After 12 months of clozapine treatment a significant volume reduction by 10% in the caudate nucleus was found. Patients who continued FGA treatment had a trend level significant increase of the caudate nucleus of 8%.

*In 1996 Frazier et al.* [44] published a longitudinal study with 8 adolescent patients (mean age 15.2 years; range: 6-18 years) with early onset schizophrenia (before the age of 12). Data on substance abuse was not provided. Before the baseline MRI scanning the patients had been medicated with FGAs for 24.8 months. Four different ROIs were assessed three of these were the caudate nucleus, putamen and globus pallidus. At baseline the volume of the caudate nucleus was significantly larger in patients than in controls, however, the volumes of putamen and globus pallidus did not differ significantly. After 24 months of clozapine treatment (mean dose 400 mg/d; range 200-600 mg/d (one patient was concomitantly treated haloperidol 1 mg/d)) the volumes of the caudate nucleus were significantly reduced in patients and no longer significanly different from controls. Likewise, the volume of the putamen decreased although not significantly so. The volume of the globus pallidus was also significantly reduced, however this was the case both in patients and in controls. The findings were not corrected for multiple comparisons. Analyses of potential associations between BG volumes, medication and clincial variables were not reported.

*In 2001 Scheepers et al.* published data on a longitudinal study with initially 28 patients (mean age 35 years; range: 19-56 years) with a diagnosis of paranoid schizophrenia. The data were presented in two separate publications after 6 and 12 months, respectively [45,46]. Substance abuse and depot medication within two months prior to the study were exclusion criteria. Patients had a mean duration of illness of 160 months and had been treated with at least one FGA for a minimum of four weeks without satisfactory clinical response (mean duration of treatment 109.6 months; mean lifetime dose of chlorpromazine equivalents 527.02 mg/d; clinical global impression (CGI) >4, severe EPS or tardive dyskinisia). The caudate nucleus was the only ROI assessed. After 6 months of clozapine treatment (mean dose 345.6 mg/d; range 200-600 mg/d) a significant reduction in the caudate nucleus volume was observed (from 8.49 cm^3^ to 8.27 cm^3^). No effect of side (left or right) was found [45]. After 12 months the reduction in the left caudate nucleus remained significant whereas the right was not. The volume reduction was driven by patients who responded to clozapine treatment (defined as a minimum of 20% reduction in PANSS total score) (n=16). No significant volumetric changes were observed in non-responderes (n=6). Across all patients (n=22) a reduction in the left caudate nucleus volume was positively correlated with improvement in PANSS total scores (driven by positive and general, but not negative symptoms). 

### 3.6 Quetiapine Monotherapy

*In 2005 Tauscher-Wisniewski et al.* [47] published a study on 27 patients with a diagnosis in the schizophrenia spectum (mean age 25.5 years). At baseline MRI all patients were antipsychotic-naïve. Data on substanse abuse was not provided. The caudate nucleus was the only ROI assessed. At baseline MRI the volume of the caudate nucleus did not signifcantly differ from a group of controls. A subgroup of patients (n=10) were subsequently treated for 3 months with quetiapine (mean dose 494 mg/d). No significant changes were observed in the volume of the caudate nucleus (baseline: 4.12 cm^3^ (left) and 4.30 cm^3^ (right); follow-up: 4.10 cm^3^ (left) and 4.19 cm^3^ (right). Analyses of potential associations between striatal volumes, medication and clincial variables were not reported.

*In 2011 Ebdrup et al.* [48] published a longitudinal study on 22 patients with a diagnosis of schizophrenia (mean age 26.2 years). At baseline MRI all patients were antipsychotic-naïve. Five patients had a lifetime, but not current diagnosis of substanse abuse. Three ROIs were assessed: the caudate nucleus, the nucleus accumbens and the putamen. Analyses of baseline striatal volumes provided in a separate publication (n=38) showed that the caudate nucleus was significantly smaller in patients than in a group of matched controls [50]. After 6 months of quetiapine treatment according to clinical need (mean dose 538 mg/d), patients had significant volume loss in caudate nucleus and putamen as compared with controls. Since a previous positon emission tomography (PET) study on the same cohort had associated a clinical effect on positive symptoms with serotonin 5-HT_2A_ receptor blockade up to 70% (corresponding to approximately 538 mg of quetiapine) [51], potential dose-dependent effects on striatum were analysed. As hypothesized, patients treated with high quetiapine doses (≥538 mg/d, n=9) had a relative volume increase as compared with patients receiving low doses of quetiapine (<538 mg/d, n=12), indirectly supporting the assumption that high quetiapine would induce more striatal D_2_ receptor blockade. Clinically, more baseline positive symptoms were associated with more volume loss in caudate nucleus. This observation appeared to be independent of the quetiapine dose, however, the correlation did not suvive a correction for multiple comparisons.

## 4 DISCUSSION

We have systematically reviewed 13 available longitudinal MRI studies on antipsychotic monotherapy investigating the effect of six different compounds on BG volumes. 

The main finding is that, contrary to our expectations, no clinical studies using antipsychotic monotherapy have shown that FGAs (specifically haloperidol and zuclopenthizol) induce significant BG volume increases. Conversely, BG volume increases have been associated with olanzapine and risperidone treatment (both classified as SGAs), although not consistently so [Table **1**]. As such, there is no evidence supporting induction of striatal volume increases as a specific feature of FGAs. Moreover, volume reductions are not restricted to SGA exposure. In general the observed effects on BG volumes were heterogeneous, e.g. increases, no changes, and decreases in BG volumes have been reported for olanzapine. An exception is clozapine which has consistently (in 3 out of 3 studies) been associated with BG volume decreases. Nonetheless, a 10-week parallel, double-blind study of patients treated with either clozapine (n=22; mean dose 414.8 mg/d) or very high doses of haloperidol (n=23; mean dose 25.4 mg/d) reported no significant differences between volumes of the caudate nucleus [52]. Numerical inspection of the baseline data (in which the patients were already chronic and medicated) indicate that the caudate nucleus volumes had further increased in the patients (n=44) [8]. (Reasons for not including the study in the present review are described in the legend to Fig. (**1**)).

In all clozapine studies the patients were chronic and treatment resistant and therefore expected to have enlarged BG volumes at inclusion. Since clozapine is not indicated for treatment of first episode psychosis [53] the effect on BG in initially antipsychotic-naïve subjects remains unknown. 

Surprisingly, 3 and 12 months treatment with haloperidol in moderate clinical doses (4.7 and 4.9 mg/d, respectively) [4] and 3 months of zuclopenthixol treatment (10.3 mg/d) [39] were not associated with significant BG increases. If indeed BG volume increase were a morphological hallmark of FGA exposure [19,28-30], we would still have expected to detect this effect. Several factors may contribute this paradox. 

First, only the FGAs haloperidol and zuclophentixol have been investigated in monotherapy studies. In two of these studies the BG volumes increased numerically, but non-significantly so [4,39]. In the remaining FGA study, 12 months haloperidol treatment was in fact associated with a marginal numerical volumetric *decreases* in the caudate nucleus and putamen, but this finding may be confounded by the fact that 8 out of 18 patients were switched to SGA treatment during the follow-up period [38]. At any rate, the volumetric in the BG increases associated with haloperidol and zuclopentixol treatment in first-episode schizophrenia patients appear marginal at best. This might imply that other compounds classified as FGAs will have more pronounced effect on BG volumes. 

Second, in the haloperidol and zuclopenthixol studies only first-episode schizophrenia patients were included. Therefore we cannot exclude other factors interacting with chronic schizophrenia and long-term antipsychotic exposure may cause BG volumes to increase, e.g. presence of EPS [12] and amphetamine abuse [54]. However, other factors associated with chronicity e.g. longer duration of illness [5], alcohol abuse [55] and possibly overweight [56], which is a common side-effect of antipsychotic drugs [33], may in fact reduce BG volumes. Thus, explanations of why monotherapy studies using FGAs fail to confirm the empirical assumption that FGAs increase BG volumes remain speculative.

Because the degree of D_2_ receptor blockade may be associated with volumetric increases in the BG [10,11,48,57,58], we *a priori* ranked the studies with respect this feature. As a result we expected volumetric increases to appear in the upper part of (Table **1**) which was not the case. Nevertheless, BG volume decreases exclusively appear in the lower part of (Table **1**). Recognizing this as a potential interaction between high D_2_ receptor and low D_2_ receptor potency, we cautiously suggest that the *absence* of a pronounced D_2_ receptor blockade (i.e. in drugs with a high k_off_ for the D_2_ receptor) may be stronger associated with BG volume decreases than the BG increases putatively associated with a pronounced D_2_ blockade. Since the natural course of the schizophrenia also appears to be associated with BG volume decreases over time [50,59,60] a weak D_2_ receptor blockade may therefore be a less disturbing intervention on brain structure. The heterogeneity of the findings could suggest that interactions with other receptor systems, e.g. the serotonin 5-HT_2A_ receptors, may obscure the volumetric effect of D_2_ receptor blockage [48,51].

### 4.1 Timing, Trajectory and Dosage

A recent study of the acute effect of haloperidol in healthy volunteers showed a reversible volumetric *decrease* in the caudate nucleus already 1-2 hours after haloperidol injection (5 mg/70 kg) [25]. This suggests that instantaneous structural brain changes following antipsychotic expose may differ from those expected after longer term treatment. By sorting the findings in columns based on duration of intervention [Table **1**] we intended to capture potential effect of the duration of treatment period on brain structure. The only pattern we prudently infer from this is, that ‘short term’ risperidone treatment (3 months) may increase BG volumes [39,40], and that this effect may be reversed after 12 months risperidone treatment [38,41,42].

Despite obvious ethical impediments of conducting withdrawal studies, one clinical study has reported decreasing volumes of nucleus accumbens and putamen after 1 year withdrawal of antipsychotic [61]. Along with the study by Tost and colleagues [25] and a preclinical study [14] current data suggest that withdrawal of antipsychotic exposure most likely is associated with BG volume decreases.

Since the dosage of antipsychotic medication may also explain the degree of BG volume changes [10,11,48,57,58] this information was included in (Table **1**). Although high doses of risperidone (6.05 mg/d) [40] and high quetiapine doses (>538 mg/d) [48] have been associated with BG increases (or less BG decreases) no clear pattern of dose-related volumetric BG changes emerges. For example, a moderate dose (14.3 mg/d) of olanzapine [43] has been associated with BG increases, whereas both lower (10.8 mg/d) [4] and higher doses (17.0 mg/d) [42] have been associated with BG decreases. The scarcity of studies which have addressed potential dose dependent effects [39,48] and the limited dose-ranges between studies compromises inferences regarding dose-related effects of specific antipsychotics on BG volumes. 

### 4.2 Clinical Relevance

Only 2 out of 13 reviewed studies reported that volumetric changes in BG correlated with clinical measures. In chronic patients treated with clozapine for 12 months, a reduction in the left caudate nucleus volume was positively correlated with improvements in PANSS total scores (driven by positive and general symptoms) [46]. In initially antipsychic-naïve patients 6 months quetiapine treatment higher positive symptoms at baseline were associated with more volume loss in caudate nucleus [48]. In both studies the findings arose from multiple comparisons why they must be interpreted cautiously. If indicative of anything, the volume of the caudate nucleus may be related to the prescence of positive symptoms. 

From a broader view the findings could imply that with optimal antipsychotic treatment, we should expect the volume of the caudate nucleus to be balanced on an ‘optimal volume’ rather than we should argue that ‘bigger is better’ or that volume loss *per se* is a measure of pathological atropy. We propose that this perspective would also be fruitful in the recent, revitalised debate of the effect of antipsychotics on global brain volumes [5,62]. 

### 4.3 Limitations

The main limitation of the current review lies within the strict inclusion criteria. Longitudinal studies investigating structural brain changes after antipsychotics monotherapy are labour demanding, expensive and therefore very difficult to conduct. Consequently, only relatively few studies have been performed and generally the sample size were small (ranging from n=8-22) with the landmark study from Lieberman and colleagues as an exception (n=71). Inherently, some studies lack statistical power and are at risk for both Type I (i.e. reporting of false positive findings) and Type II errors (i.e. reporting of false negative findings). 

The studies included in this review were published over a period of 15 years. In this period imaging techniques (e.g. stronger magnets with improved contrast, higher resolution and smaller slice thickness) and refinement of analyses (e.g. from manual ROI analyses to combinations of semi-automatised ROI analyses and tensor-based morphometry [48]) have been rapidly developed [3]. Theoretically, these methodological advances may have biased more recent studies towards Type 1 errors, whereas older studies may be biased towards Type II errors [63].

Another limitation is that our search strategy may not have been successful in identifying all relevant studies addressing our research question. To address this we also performed an unrestricted literature search as well as a manual search strategy. The three methods revealed a convincing overlap in available literature [Fig. (**1**)], why we are confident that we have covered the current topic extensively.

Finally, since a history of substance abuse was not consistently reported in the reviewed studies we cannot exclude that potential relations between substance abuse, specific antipsychotic exposure and BG volumes may account for some of the variability between studies.

### 4.4 Future Directions

Several commonly used antipsychotic compounds have not yet been used in longitudinal clinical MRI studies. Whereas previous receptor imaging studies have indicated that the optimal clinical (antipsychotic) response to D_2_ receptor blockade resides in a therapeutic window between 60-78% [64,65] the proposed relation between D_2_ receptor blockade and BG volume increases remains elusive. To minimize potentially confounding effects of other receptor systems studies treating patients with a ‘clean’ D_2_ receptor antagonist would be optimal. In this context monotherapy studies using amisulpride (a relative selective D_2_/D_3_ receptor antagonist with some serotonin 5-HT_7_ receptor affinity [66,67]) or aripiprazole (the only antipsychotic acting as a partial D_2_ receptor agonist) would be of particular interest. The application of multimodal imaging, e.g. combinations of MRI and receptor imaging techniques (PET or single-photon emission computed tomography (SPECT)) in longitudinal clinical samples would be valuable to explore associations between D_2_ receptor blockage and volumetric BG changes and their potential clinical relevance.

## CONCLUSION

Unexpectedly, no clinical studies using antipsychotic monotherapy have shown that specific FGAs induce significant BG volume increases. Conversely, both BG volumetric increases and decreases have been associated with SGA monotherapy. As such, induction of BG volume increases is not a specific feature of FGAs. Except for clozapine treatment in chronic patients, volume reductions are not restricted to specific SGAs.

The current review adds brain structural support to the notion that antipsychotics should no longer be classified as either FGAs or SGAs. Future clinical MRI studies should strive to elucidate effects of specific antipsychotic drugs. 

## Figures and Tables

**Table 1.** Dopaminergic Affinity Constants for Six Antipsychotic Compounds.

Compound	Class	Tissue	*K_i_* (n/M)
**Haloperidol**	FGA	Cloned human receptors	2.6
**Zuclopenthixol**	FGA	Rat striatum	3.0
**Risperidone**	SGA	Cloned human receptors	3.77
**Olanzapine**	SGA	Cloned human receptors	20
**Clozapine**	SGA	Cloned human receptors	210
**Quetiapine**	SGA	Cloned human receptors	770

Table 1 shows the dopaminergic (D_2_) affinity constants for the six antipsychotic compounds included in the present review. The *K_i_* value represents the amount of the antipsychotic compound (in nanomolar (n/M)) required to occupy 50% of the D_2_ receptors in vitro. Thus, a low affinity constant indicates a higher D_2_ receptor affinity. Table 1 is adapted from [31,70].

**Table 2.** Observed Volumetric Changes in Any Substructure of the Basal Ganglia After Antipsychotic Monotherapy

Time (months)*	3	6	12	24

**Haloperidol **(2 studies; 3 data points)	**n=68 ***(caudate) *(4.7 mg/d) [4]		**n=31 ***(caudate) *(4.9 mg/d) [4]	
			**n=18 ***(caudate/ putamen)* (4.9 mg/d) [38]	

**Zuclopenthixol **(1 study; 1 data point)	**n=8** *(striatum) *(10.3 mg/d) [39]			

**Risperidone **(5 studies; 5 data points)	**n=8 ***(basal ganglia) *(6.05 mg/d) [40]			
	**n=11 ***(putamen) *(3.4 mg/d) [39]			

			**n=16 ***(caudate/ putamen)* (3.7 mg/d) [38]	
			**n=15 ***(caudate/ putamen/ gl.pallidus) *(2.7 mg/d) [41]	
			**n=14 ***(caudate/ putamen/ gl.pallidus) *(2.1 mg/d) [42]	

**Olanzapine **(4 studies; 6 data points)		**n=10 ***(caudate) *(14.3 mg/d) [43]		

	**n=71 ***(caudate) *(9.5 mg/d) [4]		**n=13** *(caudate/ putamen/ gl.pallidus) *(15 mg/d) [42]	
			**n=18** *(caudate/ putamen)* (14.5 mg/d) [38]	

			**n=10 ***(putamen/ gl.pallidus)* (17.0 mg/d) [42]	
			** n=42** *(caudate)* (10.8 mg/d) [4]	

**Clozapine **(3 studies; 3 data points)		**n=26 ***(caudate) *(345.6 mg/d) [45]	**n=8 ***(caudate) *(dose: n/a) [11]	**n=8 ***(caudate/ putamen)*****(400 mg/d) [44]
			** n=22 ***(caudate) *(346 mg/d) [46]	

**Quetiapine **(2 studies; 4 data points)	**n=10 ***(caudate) *(494 mg/d) [47]	**n=9 ***(striatum)*****(≥538 mg/d) [48]		

		**n=22 ***(caudate/ putamen)*****(538 mg/d) [48]		
		**n=13 ***(caudate/ putamen)*****(<538 mg/d) [48]		

Table 2. [Light red: volume increase];[Light yellow: unchanged volume];[Light blue: volume reduction]. *(The color version of the table is available in the electronic copy of the article)*. Antipsychotic compounds are sorted based of their affinity for the dopamine D_2_ receptor as presented in Table 1. Changes are shown if only one sub-region of the basal ganglia (caudate nucleus, nucleus accumbens, putamen or globus pallidus) in a patient group significantly changed from baseline to follow-up as compared with the baseline volume or as compared with changes in a healthy control group. For positive findings (i.e. significant volume changes (red and blue cells)) the specific region(s) of interest (ROIs) which differed significantly is shown. For negative findings (i.e. unchanged volumes (yellow cells)) all investigated ROIs are shown. Both findings based on a *priori* hypotheses and exploratory analyses are shown. Significant volumetric changes which are based on the interaction between two different treatment regimens (e.g. volumetric changes in an olanzapine treated group vs. changes in a haloperidol group) are not shown in the table, but such findings are included in the text. The number of data points in the table exceeds the 13 studies included in the present review. This is because all relevant data points are displayed; i.e. studies which report on multiple MRI scannings and/or which have included different treatment arms or have investigated dose-dependent effects are displayed as individual data points.

* ’Time’ refers to time (in months) between baseline and follow-up MRI scanning.

‘n/a’: data not available.

## LIST OF ABBREVIATIONS

BG: = Basal ganglia
CGI: = Clinical global impression scale
D_2_: = Dopamine D_2_ receptor
EPS: = Extrapyramidal side-effects
FGA: = First generation antipsychotic compound
MRI: = Magnetic resonance imaging
PANSS: = Positive and negative syndrome scale
PET: = Positron emission tomography
ROI: = Region of interest
SGA: = Second generation antipsychotic compound
SPECT: = Single photon emission computed tomography
